# Anemia severity among children aged 6–59 months in Gondar town, Ethiopia: a community-based cross-sectional study

**DOI:** 10.1186/s13052-018-0547-0

**Published:** 2018-09-03

**Authors:** Mulugeta Melku, Kefyalew Addis Alene, Betelihem Terefe, Bamlaku Enawgaw, Belete Biadgo, Molla Abebe, Kindie Fentahun Muchie, Asemarie Kebede, Tadele Melak, Tsedalu Melku

**Affiliations:** 1 Department of Hematology and Immunohematology, School of Biomedical and Laboratory Sciences, College of Medicine and Health Sciences, University of Gondar, Gondar, Ethiopia; 2 Department of Epidemiology and Biostatistics, Institute of Public Health, College of Medicine and Health Sciences, University of Gondar, Gondar, Ethiopia; 3 Department of Clinical Chemistry, School of Biomedical and Laboratory Sciences, College of Medicine and Health Sciences, University of Gondar, Gondar, Ethiopia; 4 School of Nursing, College of Medicine and Health Sciences, University of Gondar, Gondar, Ethiopia; 5 School of Medicine, College of Medicine and Health Sciences, University of Gondar, Gondar, Ethiopia

**Keywords:** Anemia, Associated factors, Children, Severity

## Abstract

**Background:**

Anemia is a public health problem affecting both developed and developing countries. Childhood anemia is associated with serious consequences including growth retardation, impaired motor and cognitive development, and increased morbidity and mortality. Hence, this study aimed at assessing the prevalence and factors associated with severity of anemia among children aged 6–59 months in Gondar town, northwest Ethiopia.

**Method:**

A community-based cross-sectional study was conducted. A multi-stage sampling technique was employed to select study participants. Socio demographic and socioeconomic data were collected using a pre-tested structured questionnaire. Anthropometric measurements were taken as per WHO recommendation. Hemoglobin (Hb) concentration was measured using a portable HemoCue301 instrument (A Quest Diagnostic Company, Sweden). Mild anemia corresponds to a level of adjusted Hb of 10.0–10.9 g/dl; moderate anemia corresponds to a level of 7.0–9.9 g/dl, while severe anemia corresponds to a level less than 7.0 g/dl. Descriptive statistics were used to describe the study participants. Both bivariable and multivariable ordinal logistic regression were done, and proportional odds ratio (POR) with a 95% confidence interval (CI) was reported to show the strength of association. A *p-value* < 0.05 was considered statistically significant.

**Result:**

Out of the total of 707 children included in this study, more than half (53.5%) of them were male. The median age of children was 30 months. Two hundred two (28.6%) of children were anemic: 124(17.5%) were mildly anemic, 73(10.3%) were moderately anemic, and 5 (0.7%) were severely anemic. The young age of the child, low frequency of child complementary feeding per day, primary maternal educational status, unmarried maternal marital status, and home delivery were factors associated with severity of childhood anemia.

**Conclusion:**

Anemia among children aged 6–59 months in Gondar Town was a moderate public health problem. Improving access to education, providing regular health education about childcare and child feeding practices, strengthening the socioeconomic support for single-parent families and conducting regular community-based screening are recommended to reduce childhood anemia.

## Background

Anemia, defined as a low blood hemoglobin (Hb) concentration, is one of the most common and widespread disorders in the world, affecting one-quarter of the world’s population. It is a major public health problem in several countries, particularly common among preschool-aged children and women [1]. According to the 2011 World Health Organization (WHO) report, anemia resulting from iron deficiency was one of the most important factors contributing to the global burden of diseases, and it increases morbidity and mortality in preschool-aged children and pregnant women [2]. Globally, anemia affects 1.62 billion people, which correspond to 24.8% of the population. The highest prevalence is found in preschool-age children (47.4%). WHO regional estimate indicated that the highest proportion of anemic preschool-age children are residing in Africa [2, 3]. Anemia is also a major public health problem among preschool-aged children in Ethiopia [4].

Childhood anemia has a substantial association with social, economic, psychological, and health-related problems. The evidence demonstrated that early childhood anemia is a strong predictor of adulthood anemia [4]. A considerable number of studies reported that childhood iron deficiency anemia has been strongly correlated with psychiatric disorders, mental retardation and developmental disorders [5–8]. Anemia dysfunctions recent and past memory concentration domain of the children. Children with anemia have a lower developmental index, poor motor development, no interest in their environment, a shorter attention span, diminished cognitive ability and behavioural problems [9]. As a result, anemia at early childhood continues to affect school achievement and behavioural development negatively [10, 11]. In addition, childhood anemia is a common condition causing significant morbidity and mortality. Severe anemia carries a high ‘hidden’ morbidity and mortality occurring months after initial diagnosis and treatment, and it is the common contributing factor for overall under-five mortality [12].

The etiology of anemia is often multi-factorial and interrelated in a complex way. In developing countries, micronutrient deficiency and infectious diseases take the greatest part [13–17]. Socio-economic status, demographic characteristics, child feeding practices, accessibility for preventive and curative health services have also been playing a major role [18–20]. Maternal anemia during pregnancy may also be associated with the development of childhood anemia [21, 22]. Besides, genetic factors like hemoglobinopathies are the cause of anemia [14, 23].

Anemia prevalence data remains to be an important indicator of public health since anemia is related to morbidity and mortality, particularly in a most vulnerable segment of the population such as preschool-aged children and pregnant women [22]. In order to make a full use of these prevalence data, information on factors associated with anemia should be collected so that interventions can be better adapted to the local situation and can, therefore, be more effective. However, much is not known about the prevalence and associated factors of childhood anemia severity in Ethiopia. Thus, the aim of this study was to assess the prevalence and factors associated with severity of anemia among children aged 6–59 months in Gondar town, northwest Ethiopia.

## Methods

### Study design, population, and sampling technique

A community-based cross-sectional study was conducted in April 2015 among children aged 6–59 months in Gondar town, northwest Ethiopia. A sample size of 735 was calculated by single population proportion formula, considering estimated prevalence of anemia (35.1%) among children aged 6–59 month in Amhara region [4], 95%CI, 5% margin of error, design effect of 2, and 5% non- response rate. A multi-stage random sampling technique was employed to select study participants in two stages. At the first stage, four out of twelve *kebeles* (smallest administrative units) (i.e. 30% of the total area) were selected by simple random sampling technique. At the second stage, a total of 735 households were selected using a systematic random sampling method with proportional allocation to each selected *kebeles*. The total number of households with children aged between 6 and 59 months was obtained from the respective administrative areas and used to calculate the sampling fraction. In the case where more than one children were found eligible in the selected households, only one of them was chosen randomly using the lottery method.

### Data collection methods and instruments

A pretested structured questionnaire was used to collect socio-demographic and economic data from mothers by face-to-face interview. Anthropometric measurements such as weight and height were measured for children according to the 2006 WHO recommendation [24]. Z-scores for weight-for-age (WAZ), height-for-age (HAZ), and weight-for-height (WHZ) were calculated using WHO Anthro software. Nutritional status was defined as underweight if WAZ was less than − 2 standards deviation (SD), stunting if HAZ was less than -2SD, and wasting if WHZ was less than -2SD [25]. Body mass index (BMI) was also calculated for the mothers according to the WHO STEP-wise surveillance manual [26].

Hb was measured by a portable HemoCue301 instrument (A Quest Diagnostic Company, Sweden) from capillary blood. HemoCue method of Hb determination is recommended by WHO to determine population prevalence of anemia, and several studies have established the validity of this instrument [24, 27]. After adjusting Hb concentration for altitude, anemia was defined as mild if Hb was between 10 and 10.9 mg/dl, moderate if between 9.9 and 7 g/dl, and severe if < 7 g/dl [24].

### Data quality assurance

The questionnaire was prepared in English, translated to Amharic and then translated back to English to check for consistency. Data were collected by trained data collectors (BSC nurses and senior medical laboratory technologists) after training was given about the objective of the study, confidentiality issues, study participants’ right, consenting, techniques of interview, and Hb and anthropometric measurements. The data collection process was closely supervised by investigators. All measurements were performed by following the manufacturers’ recommendation.

### Data management and analysis

Data were entered using Epi Info version 3.5.3 statistical software, and then exported to SPSS version 20 for analysis. Descriptive statistics including frequencies, percentages, median, and interquartile range were performed to describe the study participants. The bi-variable and multi-variable proportional odds model (POM), the most widely used family of ordinal logistic regression in epidemiological studies, was fitted to identify factors associated with severity of childhood anemia. The proportionality assumptions for POM were checked using Chi-square parallel line tests, (*p-value* = 0.791) indicating that the assumption was not violated. The Pearson chi-square goodness-of-fit test showed that the model fitted the data well (*p* = 0.152). All variables with a *p-value* ≤ 0.2 in the bivariable analysis were fitted into the multivariable analysis to control confounding effects. Adjusted proportional odds ratio (aPOR) with a 95% CI was used to evaluate the strength of statistical association between explanatory and outcome variables. All variables with *p-value*s < 0.05 in the multi-variable analysis were considered to be statistically significant.

## Result

### Characteristics of study participants

A total of 735 children were selected; of whom 707 participated in the study, with a response rate of 96.2%. More than half, 378 (53.5%), of the children were male. The median age of children was 30 months (interquartile range (IQR) =24 months). From the total children, 323 (45.7%) were stunted, 562 (79.5%) were delivered at health institutions, and 577(81.6%) had been exclusively breastfed for 6 months (Table 1).

**Table 1:** Characteristics of children aged 6–59 months who participated in the study, Gondar Town, April 2014

Characteristics	Categories	Frequency (n)	Percentage (%)
Sex of child	Male	378	53.5
	Female	329	46.5
Age of child (months)	6–11	74	10.5
	12–23	181	25.6
	24–35	174	24.6
	36–47	155	21.9
	48–59	123	17.4
Child’s place of delivery	At home	145	20.5
	At health institution	562	79.5
Child’s birth type	Singleton	692	97.9
	Twins	15	2.1
Mode of delivery	CS or instrumental delivery	73	10.3
	spontaneous vaginal delivery	634	89.7
Pre-lacteal feeding	Yes	58	8.2
	No	649	91.8
Exclusive breastfeeding status^a^	Exclusively breastfed	577	81.6
	Not exclusively breastfed	130	18.4
Breastfeeding status	Being breastfed	301	42.6
	Not being breastfed	406	57.4
Preceding birth interval^b^	12–24 months	83	19.9
	25–48 months	149	35.6
	> 48 months	186	44.5
WHZ	Wasted	46	6.5
	Normal	661	93.5
HAZ	Stunted	323	45.7
	Normal	384	54.3
WAZ	Underweight	111	15.7
	Normal	596	84.3
Complementary feeding frequency	< 4 time per day	210	29.7
	4–5 times per day	440	62.2
	> 5 time per day	57	8.1

^a^An exclusive breastfeeding time was until 6 months of age as to WHO standard; ^b^Percentage was calculated for 418 children whose birth order is 2nd and above; CS: Caesarian section; HAZ: Height-for-Age Z-score; WAZ: Weight-for-Age Z-score; WHZ: Weight-for-Height Z-score

### Parental characteristics

Concerning the maternal characteristics, 619(87.6%) were married, 500(70.7%) were housewives, 472(66.8%) had a normal weight, and 364(51.5%) were 25–30 years old. About 287(40.6%) of children’s fathers were governmental or private sector employees, and 237(33.5%) attended primary education. More than half, 376(53.2%), of children were living in a family with a monthly income of 60–124 US dollar ($). Moreover, 143(20.2%) of the children were living in a family with more than five members, and 142(20.1%) were living in a family with two or more under five-year-old children (Table 2).

**Table 2:** Socio-demographic and economic characteristic of parents of children participated in the study, Gondar Town, April 2014

Characteristics	Categories	Frequency (n)	Percentage (%)
Maternal age(year)	< 25	165	23.3
	25–30	364	51.5
	31–35	102	14.4
	> 35	76	10.7
Maternal marital status	Married	619	87.6
	Unmarried^a^	88	12.4
Maternal educational status	No formal education	220	31.1
	Primary education	210	29.7
	Secondary education	218	30.8
	Tertiary education	59	8.9
Maternal occupation	Housewives	500	70.7
	Government or private sector employees	38	5.3
	Merchants	58	8.2
	Daily laborer	111	15.8
Maternal BMI	Underweight	95	13.4
	Normal weight	472	66.8
	Overweight	140	19.8
Paternal educational status	No formal education	163	23
	Primary education	237	33.5
	Secondary education	220	31.1
	Tertiary education	87	12.3
Paternal occupation	Government or private sector employees	287	40.6
	Merchants	236	33.4
	Daily laborer	184	26
Family size	< 4	250	35.4
	4–5	314	44.4
	> 5	143	20.2
Number of under-five children	1	565	79.9
	≥2	142	20.1
Family monthly income	< $60	162	22.9
	$60–124	376	53.2
	> $124	169	23.9

^a^includes single and widowed**,** 1USD is equivalent to 20.90 Ethiopian Birr based on the 2015 exchange rate; Unmarried^a^: includes Single, divorced and widowed; BMI: Body Mass Index

### Prevalence of anemia

The overall prevalence of anemia among children aged 6–59 months was 202 (28.6%) (95% CI: 25.2–31.9%). The prevalence of mild, moderate, and severe anemia were 124(17.5%), 73(10.3%), and 5(0.7%), respectively. The highest prevalence of anemia (46.8%) was found in children whose mothers did not receive antenatal care (ANC) during pregnancy period of sampled children. Regarding the severity, the highest prevalence of severe anemia (6.7%) was found in twin children. About 24.3%, 21.6% and 2.7% of children aged 6–11 months were mildly, moderately and severely anemic, respectively. Out of the total children being breastfed during the data collection, 1.3%, 15.9% and 23.6% of them were severely, moderately and mildly anemia, respectively (Table 3).

**Table 3:** The prevalence and severity of anemia according to selected children and parental characteristics

Variable	Categories	Anemic status and severity level (n (%))				
		Severely anemic	Moderately anemic	Mildly anemic	Non-anemic	Overall anemia prevalence
Sex of child	Male	3 (0.8)	38 (10.1)	69 (18.3)	268 (70.9)	110 (29.1)
	Female	2 (0.6)	35 (10.6)	55 (16.8)	237 (72.0)	92 (28.0)
Age of child (months)	6–11	2 (2.7)	16 (21.6)	18 (24.3)	38 (51.4)	36 (48.6)
	12–23	1 (0.6)	29 (16.0)	45 (24.9)	106 (58.8)	75 (41.4)
	24–35	1 (0.6)	19 (10.9)	27 (15.5)	127 (73.0)	47 (27.0)
	36–47	1 (0.6)	6 (3.9)	26 (16.8)	122 (78.7)	33 (21.3)
	48–59	0	3 (2.4)	8 (6.5)	112 (91.1)	11 (8.9)
WHZ	Wasted	0	6 (13.0)	8 (17.4)	32 (69.6)	14 (30.4)
	Normal	5 (0.8)	67 (10.1)	116 (17.5)	473 (71.6)	188 (28.4)
HAZ	Stunted	3 (0.9)	30 (9.3)	60 (18.6)	230 (71.2)	93 (28.8)
	Normal	2 (0.5)	43 (11.2)	64 (16.7)	275 (71.6)	109 (28.4)
WAZ	Underweight	2 (1.8)	9 (8.1)	23 (20.7)	77 (69.4)	34 (30.6)
	Normal	3 (0.5)	64 (10.7)	101 (16.9)	428 (71.8)	168 (28.2)
Maternal age	< 25 years	1 (0.6)	18 (10.9)	41 (24.8)	105 (63.6)	60 (36.4)
	25–35 years	0	7 (9.2)	13 (17.1)	56 (73.7)	20 (26.3)
	> 35 years	4 (0.9)	48 (10.3)	70 (15.0)	344 (73.8)	122 (26.2)
Maternal education	No formal education	2 (0.9)	25 (11.4)	42 (19.1)	151 (68.6)	69 (31.4)
	Primary education	0	27 (12.9)	44 (21.0)	139 (66.2)	71 (33.8)
	Secondary and above	3 (1.1)	21 (7.6)	38 (13.7)	215 (77.6)	62 (22.4)
Frequency of ANC visits	Never visited	1 (2.1)	9 (9.1)	12 (25.5)	25 (53.2)	22 (46.8)
	1–4 times visited	4 (0.7)	55 (9.4)	101 (17.2)	428 (72.8)	160 (27.2)
	More than 4 times visited	0	9 (12.5)	11 (15.3)	52 (72.2)	20 (27.8)
Child’s place of delivery	At home	1 (0.7)	25 (17.1)	29 (20.0)	90 (62.1)	55 (37.9)
	At health institution	4 (0.7)	48 (8.5)	95 (16.9)	415 (73.8)	147 (26.2)
Type of child birth	Twin	1 (6.7)	4 (26.7)	3 (20.0)	7 (46.7)	15 (53.3)
	Singleton	4 (0.6)	69 (10.0)	121 (17.5)	498 (72.0)	194 (28.0)
Frequency of Complementary feeding	< 3 times per day	1 (3.7)	6 (22.2)	7 (17.1)	13 (64.8)	14 (519)
	3–4 times per day	3 (0.6)	54 (10.0)	90 (16.6)	394 (72.8)	147 (27.2)
	> 4 times per day	1 (0.7)	13 (9.4)	27 (19.4)	98 (70.5)	41 (29.5)
Loss of appetite	Yes	2 (1.4)	15 (10.7)	34 (24.3)	89 (63.6)	51 (36.4)
	No	3 (0.5)	58 (10.2)	90 (15.9)	416 (73.4)	51 (9.0)
Presence of morbidity symptoms	Yes	2 (1.3)	16 (10.5)	38 (25.0)	96 (63.2)	56 (36.8)
	No	3 (0.5)	57 (10.3)	86 (15.5)	409 (73.7)	146 (26.3)
Child being breastfed	Yes	4 (1.3)	48 (15.9)	71 (23.6)	178 (59.1)	123 (40.9)
	No	1 (0.2)	25 (6.2)	53 (13.1)	327 (80.3)	79 (19.5)
Presence of Diarrhea	Yes	0	8 (9.6)	23 (27.1)	52 (62.7)	31 (37.3)
	No	5 (0.8)	65 (10.4)	101 (62.2)	453 (72.6)	624 (27.4)
Preceding birth interval^a^	12–24 months	1 (1.2)	9 (10.8)	16 (19.3)	57 (68.7)	26 (31.3)
	25–48 months	1 (0.7)	19 (12.8)	24 (16.1)	105 (70.5)	44 (29.5)
	> 48 months	1 (0.5)	16 (8.6)	26 (14.0)	143 (76.9)	43 (23.1)
Maternal marital status	Married	4 (0.6)	61 (9.9)	106 (17.1)	448 (72.4)	171 (27.6)
	Unmarried^c^	1 (1.1)	12 (13.6)	18 (20.5)	57 (64.8)	31 (35.2)
Pre-lacteal feeding	Yes	0	8 (13.8)	13 (22.4)	37 (63.8)	21 (36.2)
	No	5 (0.8)	65 (10.0)	111 (17.1)	468 (72.1)	181 (27.9)
Number of under five children	1	2 (0.4)	58 (10.3)	100 (17.7)	405 (71.7)	160 (28.3)
	≥2	3 (2.1)	15 (10.6)	24 (16.9)	100 (70.4)	42 (29.6)
Family monthly income^b^	< 108$	4 (0.9)	50 (11.2)	88 (19.7)	304 (68.2)	142 (31.8)
	≥ 108$	1 (0.4)	23 (8.8)	36 (13.8)	201 (77.0)	60 (23.0)

*ANC* antenatal care; ^a^Percentage was calculated for 418 children whose birth order is 2nd and above. ^b^classified on the basis of the mean value; Unmarried^c^: includes Single, divorced and widowed

### Factors associated with anemia

In the bivariable POM, age of child, maternal age, maternal educational status, maternal marital status, ANC visit at that time of pregnancy, child’s place of birth(home delivery), frequency of complementary feeding per day, loss of appetite, presence of morbidity symptoms (fever, vomiting, fast breathing and cough), breastfeeding status at the time of survey, and family monthly income were associated with anemia severity. However, in multivariable POM, being a young aged child (5–11 months [aPOR =13.9, 95%CI: 3.50–35.02], 12–23 months [aPOR = 8.53, 95%CI: 3.8–19.18], 24–35 months [aPOR = 4.77, 95%CL: 1.67–10.04], 36–47 months [aPOR = 3.58, 95%: 1.67–7.65]), being child of a mother with a primary educational status [aPOR = 1.71, 95%CI: 1.10–2.65], being a child delivered at home [aPOR = 1.64, 95%CI: 1.03–2.61], being child of mother whose marital status was unmarried [aPOR = 1.8, 95%CI: 1.07–3.03], and low frequency of complementary feeding practice per day [aPOR = 2.46, 95%CI: 1.02–5.77] were the factors associated with childhood anemia severity (Table 4).

**Table 4:** Factors associated with childhood anemia severity in multivariable POM considering anemic status as outcome variable with three ordered categories

Variable	Categories	Anemic status and severity level(n(%))			Bivariable POM Analysis		Multivariable POM analysis	
		Severe or moderate anemia (n(%))	Mild anemia (n(%))	No anemia	cPOR (95%CI)	*p-value*	aPOR (95%CI)	*p-value*
Age of child (months)	6–11	18 (24.3)	18 (24.3)	38 (51.4)	10.20 (4.81, 21.71)	< 0.001	13.9 (5.5, 35.02)	< 0.001
	12–23	30 (16.6)	45 (24.9)	106 (58.6)	7.17 (3.56, 14.24)	< 0.001	8.53 (3.8, 19.18)	< 0.001
	24–35	20 (11.5)	27 (15.5)	127 (73.0)	3.88 (1.92, 7.82)	< 0.001	4.77 (1.67, 10.04)	< 0.001
	36–47	7 (4.5)	26 (16.8)	122 (78.7)	2.67 (1.29, 5.55)	0.008	3.58 (1.67, 7.65)	0.001
	48–59	3 (2.4)	8 (6.5)	112 (91.1)	1.00		1.00	
Maternal age	< 25 years	19 (11.5)	41 (24.8)	105 (63.6)	1.51 (1.04, 2.19)	0.032	0.94 (0.60, 1.47)	0.7794
	> 35 years	7 (9.2)	13 (17.1)	56 (73.7)	0.98 (0.57, 1.69)	0.943	0.92 (0.49, 1.74)	0.806
	25–35 years	52 (11.2)	70 (15)	344 (73.8)	1.00		1.00	
Maternal education	No formal education	27 (12.3)	42 (19.1)	151 (68.6)	1.57 (1.06, 2.34)	0.025	1.19 (0.72, 1.96)	0.489
	Primary education	27 (12.9)	44 (21)	139 (66.2)	1.74 (1.17, 2.59)	0.006	1.71 (1.10, 2.65)	0.017
	Secondary and above	24 (8.7)	38 (13.7)	215 (77.6)	1.00		1.00	
ANC visits	Never visited	10 (21.3)	12 (25.5)	25 (53.2)	2.23 (1.06, 4.69)	0.035	1.42 (0.59, 3.39)	0.433
	1–4 time visited	59 (10.0)	101 (17.2)	428 (72.8)	0.94 (0.55, 1.61)	0.831	0.72 (0.39, 1.31)	0.281
	More than 4 times visited	9 (12.5)	11 (15.3)	52 (72.2)	1.00		1.00	
Child’s birth place	At home	26 (17.9)	29 (20.0)	90 (62.1)	1.8 (1.23, 11.99)	0.002	1.64 (1.3, 2.61)	0.038
	At health institution	52 (9.3)	95 (16.9)	145 (73.8)	1.00		1.00	
Frequency of Complementary feeding	< 3 times per day	7 (25.9)	7 (25.9)	13 (48.1)	2.68 (1.22, 5.92)	0.015	2.46 (1.02, 5.77)	0.003
	3–4 times per day	57 (10.5)	90 (16.9)	394 (72.8)	0.91 (0.61, 1.37)	0.853	1.51 (0.94, 2.43)	0.181
	> 4 times per day	14 (10.1)	27 (19.4)	98 (70.5)	1.00		1.00	
Loss of appetite	Yes	17 (12.1)	34 (24.3)	89 (63.6)	1.51 (1.02, 2.20)	0.039	1.14 (0.74, 1.75)	0.554
	No	61 (10.8)	90 (15.9)	416 (73.4)	1.00		1.00	
Presence of morbidity symptom	Yes	18 (11.8)	38 (25.0)	96 (63.2)	1.54 (1.06, 11.60)	0.025	1.46 (0.83, 2.58)	0.191
	No	60 (10.8)	86 (15.5)	409 (73.7)	1.00		1.00	
Child being breastfed	Yes	52 (17.3)	71 (23.6)	178 (59.1)	2.88 (2.08, 4.01)	< 0.001	1.24 (0.76, 2.03)	0.382
	No	26 (6.4)	53 (13.1)	327 (80.5)	1.00		1.00	
Presence of Diarrhea	Yes	8 (9.6)	23 (27.7)	52 (62.7)	1.43 (0.89, 2.30)	0.135	0.64 (0.31, 1.31)	0.222
	No	70 (11.2)	101 (16.2)	453 (72.6)	1.00		1.00	
Preceding birth interval	12–24 months	10 (12)	16 (19.3)	57 (68.7)	1.04 (0.62, 1.75)	0.882	1.0 (0.52, 1.92)	0.9963
	25–48 months	20 (13.4)	24 (16.1)	105 (70.5)	0.99 (0.65, 1.51)	0.949	0.76 (0.42, 1.35)	0.346
	> 48 months	17 (9.1)	26 (14.0)	143 (76.9)	0.69 (0.46, 1.05)	0.087	0.71 (0.40, 1.24)	0.231
	First child	31 (10.7)	58 (20.1)	200 (69.1)	1.00		1.00	
Maternal marital status	Unmarried*	13 (14.8)	18 (20.5)	57 (64.8)	1.43 (0.90, 2.28)	0.126	1.8 (1.07, 3.03)	0.026
	Married	65 (10.5)	106 (17.1)	448 (72.4)	1.00		1.00	
Pre-lactal feeding	Yes	8 (13.8)	13 (22.4)	37 (63.8)	1.44 (0.83, 2.50)	0.194	1.33 (0.71, 2.46)	0.37
	No	70 (10.8)	111 (17.1)	468 (72.1)	1.00		1.00	
Family monthly income	<$108	54 (12.1)	88 (19.7)	308 (68.2)	1.54 (1.09, 2.18)	0.015	1.35 (0.91, 2.01)	0.136
	≥$108	24 (9.2)	36 (13.8)	201 (77.0)	1.00		1.00	
Family size	> 5	17 (11.9)_	26 (18.2)	100 (69.9)	0.95 (0.61, 1.47)	0.819	1.51 (0.83, 2.77)	0.179
	4–5	33 (10.5)	47 (15.0)	234 (74.5)	0.84 (0.53, 1.10)	0.145	1.16 (0.71, 1.90)	0.562
	< 4	28 (11.2)	51 (20.4)	171 (68.4)	1.00		1.00	

The outcome variable, anemia, was classified in three ordered categories as 0 = no anemia, 1 = Mild anemia, and 2 = Moderate or Severe anemia; The reference category of each independent variables was set last during analysis; aPOR: adjusted Proportional Odds Ratio; cPOR: crude Proportional Odds Ratio; CI: confidence Interval; POM: Proportional Odds Model; Unmarried*: includes single, divorced and widowed

Model fitness information: Test of parallel lines for the proportional odds assumption: Chi-square = 19.123, df = 25, *p-value* = 0.791; Goodness-of-fit test of overall model: Pearson Chi-square = 1275.797, df = 1225, *p-value* = 0.152, Pseudo R^2^ = 0.182

## Discussion

Anemia prevalence data remain to be an important indicator of public health since anemia is related to morbidity and mortality, particularly in more vulnerable -preschool aged children and pregnant women [22]. As per WHO and United Nations report on the progress of achieving Millennium development goals (MDG), even though substantial progress has been made towards achieving MDG4 to reduce the number of under-five mortality rate worldwide, the rate of decline remains insufficient to meet the stated goal, particularly in sub-Saharan Africa and southern Asia. By the year 2011, children born in sub-Saharan Africa faced a higher probability of dying before the age of five than children born elsewhere [28, 29]**.** This raises questions about the impact and effectiveness of interventions made to reduce the burden of anemia, since anemia prevalence is a useful indicator to assess the impact and effectiveness of interventions [22].

In this study, the overall prevalence of anemia among children aged 6–59 months was found to be 28.5%. It is a moderate public health problem and should be addressed using appropriate intervention strategies since anemia contributes to childhood morbidity and mortality [24]. This prevalence is consistent with studies done in South-central Ethiopia [30], Timor-Leste [18], and Northeastern Brazil [31]. However, it is lower than studies conducted in another part of Ethiopia [32–34]. Tanzania [35], Benin and Mali [36], Haiti [37], Bangladesh [38], Indonesia [17], and Pernambuco, Northeastern Brazil [39]. In contrary to this, it is higher than studies conducted in Acrelandia, Western Brazilian Amazonia [19], and Vitoria, Brazilian [40]. The plausible reasons for disparities in anemia prevalence between the present study and aforementioned studies might be related to the seasonal and geographic variability of risk factors, and differences in socioeconomic status of the populations in which the studies were conducted.

In our study, the prevalence of mild anemia was higher than the other types of anemia. This result is consistent with previous studies conducted in Northern Ethiopia [33], Haiti [37], and Western China [41], in which mild anemia was reported as the most common type of anemia among children. This could be due to the fact that children with mild anemia are mostly asymptomatic, and they may not seek medical intervention and may not get treatment. The clinical symptoms may not be presented in children with mild anemia, as the body often compensates for the gradual changes in Hb concentration.. This indicates that anemia is a hidden public health problem that affects a significant number of children. The other possible reason could be due to the fact that almost half of the children in our study were stunted, where mild anemia is more common in stunted children.

In this study, younger aged children were more likely to be anemic. The likelihood of being anemic among children aged 6–11 months was higher as compared to those who were within the age of 48–59 months. This is in agreement with other studies [34, 42]. Besides, the odds of being anemic were higher for children who were in the age group of 6–23 months, which is consistent with studies done elsewhere [19, 33, 37, 39, 43]. The reasons for a high likelihood of being anemic in younger aged children may be due to different factors. The first reason would be a nutritional imbalance, as young children require relatively large nutritional demands owing to the high rate of growth during the first two years of life in combination with rapid expansion of blood volume. Since the practices and timely initiation of complementary feeding is poor in Ethiopia as evidenced by Ethiopian Demographic and Health Survey [44], younger children may suffer from at least one micronutrient deficiencies (Vitamin B12, folate, or iron) which leads to the onset of anemia. The second reason may be due to the low concentration of iron and other micronutrients in breast milk that cannot be sufficient for daily requirement of child growth [45]. Thirdly, it would also be due to the high susceptibility of young children to infectious diseases, which affects the absorption and utilization of bioavailable of micronutrients. Although our study did not include infectious diseases such as malaria and intestinal parasitic infection, they are highly prevalent in Gondar town [46, 47], and may also be a cause for red blood cell hemolysis and loss of appetites, which exacerbate the problem [48].

Consistent with previous studies [18, 22, 49], our study showed that older children were less likely to be anemic as compared to younger aged children As described in previous research [39], the Hb concentration had a linear and a positive association with age. The possible explanation for this may be due to the fact that with an increase in age, the nutritional demand for growth relatively becomes lower than early age. In addition, the children tend to discontinue breastfeeding and get involved in complementary feeding so that they can eat more varied diet [50]. Thus, as the children getting older, they become less likely to be anemic.

Children of mothers with primary education were two times more likely to be anemic as compared to children whose mothers attended secondary and/or above education. The finding is consistent with the literature [14, 49, 51]. This may be related to the knowledge and practices of mothers about child feeding and health care; mothers with low educational level may not have adequate knowledge regarding the appropriate child health care practices and feeding [31]. Moreover, low level of maternal education may have a negative impact on the socioeconomic status of the family, which would affect the child nutritional status and optimal childcare [3, 43].

In this study, children who had a low frequency of complementary feeding practices of less than three times per day were at a greater risk of being anemic as compared to those who had a practice of feeding five or more times per day. This is consistent with previous studies [52–54], revealing that low frequency of child complementary feeding practice increased the risk of anemia. After six months, the introduction of complementary feeding is a recommended practice to adequately support the daily nutritional requirement of children. However, the practices of timely introduction of complementary foods were reported to be poor in Ethiopia, which could contribute to the low body iron level in children and ultimately causes a higher prevalence of childhood anemia [44]. Moreover, since the iron concentration in breast milk is low, insufficient to meet the daily requirement for normal children’s physiological activities as well as due to the high prevalence of maternal micronutrient deficiencies in developing countries [43], consumption of balanced complementary foods which meets the minimum dietary diversity and frequency is mandatory for children [1].

With regard to maternal marital status, children of unmarried mothers (i.e. single, widowed and divorced) were more likely to be anemic as compared to those whose mothers were married. Evidence speculated that parental marital status had an impact on family structure and socioeconomic status [55, 56]. The economic deprivation of unmarried mother may be partially responsible for the disadvantaged socioeconomic outcomes as well as the well-being of children. Moreover, children whose parents lack financial resources are less likely to receive high-quality of child care, health care and other social services [26]. Low socioeconomic status may, in turn, increase the risk of food insecurity, malnutrition and susceptibility to infectious diseases, which inevitably results in the development of childhood anemia [25]. Furthermore, evidence revealed that unmarried mothers experience a high rate of major depressive illness and distress [28], and they also spend less time with their children than married mothers do [57]. Therefore, low socioeconomic status and depression illness in unmarried mothers would likely influence the quality of child care that they provide for their children, and this may be the cause of childhood anemia.

In this study, a place where a child was delivered has been significantly associated with severity of childhood anemia. In children who were delivered at home, the odds of being anemic was 1.64 times higher than those children who were delivered at a health institution. Institutional delivery improves maternal health as well as healthcare practices related to infant and child care. Contrary to this, home delivery has been implicated in maternal and child morbidity and mortality. Evidence revealed that delayed breastfeeding beyond two hours and pre-lacteal feeding were high in children who were delivered at home than health institution [58]. Delayed breastfeeding initiation and pre-lactal feeding increase the risk of child morbidity [59, 60], and thus contribute to anemia development.

### Limitations

This study has some limitations. Firstly, the cross-sectional nature of the study design does not allow to establish a cause-and-effect relationship. Secondly, this study did not include all modifiable risk factors as well as common infectious diseases that potentially deregulate the hematopoiesis such as HIV intestinal parasite, and malaria. Thirdly, we used only Hb value to define anemia, and serum hematinic levels were not assessed. Despite these limitations, it is the first community-based cross-sectional study, which tried to show the prevalence and associated factors of childhood anemia severity in the study area.

## Conclusions

In conclusion, anemia was found to be a moderate public health problem among children aged 6–59 months in Gondar Town. The study revealed that young child age, primary maternal educational level, low frequency of child complementary feeding practices, home delivery and unmarried maternal marital status (i.e. single, divorced and widowed) were factors associated with childhood anemia severity in the study setting. Therefore, appropriate and tailored interventional strategies are required to reduce the prevalence of childhood anemia. These include improving women’s access to education; providing health education on child feeding practices; and strengthening nutritional and social supports. Furthermore, further in-depth studies need to be conducted using a large sample size and including assessment of serum micronutrient level.

## Abbreviations

ANC: Antenatal care
aPOR: adjusted Proportional Odds Ratio
BMI: Body Mass Index
CI: Confidence Interval
cPOR: crude Proportional Odds Ratio
CS: Caesarian Section
HAZ: Z-score for Height-for-Age
Hb: Hemoglobin
IQR: Interquartile Range
MDG: Millennium Development Goals
POM: Proportional Odds Model
SD: Standard Deviation
USD: US Dollar
WAZ: Z-scores for Weight-for-Age
WHO: World Health Organization
WHZ: Z-score for Weight-for-Height
